# Improved Dielectric Properties of Thermoplastic Polyurethane Elastomer Filled with Core–Shell Structured PDA@TiC Particles

**DOI:** 10.3390/ma13153341

**Published:** 2020-07-27

**Authors:** Xinfu He, Jun Zhou, Liuyan Jin, Xueying Long, Hongju Wu, Li Xu, Ying Gong, Wenying Zhou

**Affiliations:** 1 School of Chemistry and Chemical Engineering, Xi’an University of Science and Technology, Xi’an 710054, China; zj18209251217@126.com (J.Z.); jinliuyan0423@163.com (L.J.); echo_islxy@163.com (X.L.); wuhongju2012@xust.edu.cn (H.W.); xustxuli2016@163.com (L.X.); gongying0311@163.com (Y.G.); 2 Key Laboratory of Coal Resources Exploration and Comprehensive Utilization, Ministry of Natural Resources, Xi’an 710021, China

**Keywords:** polymer matrix composites, electrical properties, mechanical properties, polyurethane, titanium carbide, polydopamine

## Abstract

Insulating interlayer between nanoparticles and polymer matrix is crucial for suppressing the dielectric loss of polymer composites. In this study, titanium carbide (TiC) particles were surface modified by polydopamine (PDA), and the obtained PDA@TiC powders were used to reinforce thermoplastic polyurethane (TPU). The results indicate that the PDA@TiC were homogenously dispersed in the matrix compared with the pristine TiC, and that the PDA@TiC/TPU composites show improved dielectric and mechanical properties, i.e., much lower dissipation factors and obviously enhanced dielectric breakdown strength, as well as higher tensile strength and elongation at break as compared to the raw TiC/TPU. The nanoscale PDA interlayer contributes to the dielectric and mechanical enhancements because it not only serves as an insulating shell that prevents TiC particles from direct contacting and suppresses the loss and leakage current to very low levels, but also enhances the interfacial interactions thereby leading to improved mechanical strength and toughness. The prepared flexible PDA@TiC/TPU with high permittivity but low loss will find potential applications in electronic and electrical applications.

## 1. Introduction

Polymer-based dielectric composites have aroused great interest in researchers due to the materials being able to store electrical energy and their wide use in capacitors, voice control equipment, artificial muscles and other applications [1,2,3,4]. However, the dielectric constant (*k*) of most commercialized polymers are often very low (*k* < 10), which cannot meet the requirements of further miniaturization of electronic components in practical applications. Studies have shown that direct introduction of inorganic particles with high-*k*, such as barium titanate, calcium titanate and piezoelectric ceramics, can significantly increase the dielectric constant of polymer matrix composites [5,6,7,8,9]. Among all the electroactive polymers, thermoplastic polyurethane (TPU) has been regarded as a good flexible polymer host owing to its excellent electrical properties, high shape recoverability, and large deformation. Yuan et al. [10] prepared a novel TPU based composite and found that the dielectric constant of TPU increased from 8 to 41 with the incorporation of 1.0 wt % polyamide-1 (PA1), the loss tangent still kept at a low level of less than 0.02. Zheng et al. [11] blended TPU with poly (vinylidene fluoride) (PVDF) and found the soft chain of TPU was very helpful for promoting the breakdown strength of composites, and a maximum value was obtained of up to 537.8 mV/m at 3 vol.% TPU with a hardness of 65. Xiao et al. [12] prepared a series of TPU/rGO (reduced graphene oxide) composites with high dielectric permittivity (151 at 1 kHz) with only 0.75 vol.% rGO. Meanwhile, its dissipation factors remain lower than 1 at 1 kHz. Chen et al. [13] synthesized poly (methyl methacrylate)-functionalized graphene/polyurethane (MG-PU) dielectric elastomer composites, and found the 1.5 wt % MG-PU film had high relative permittivity as up to 28.2.

Titanium carbide (TiC) as a structural ceramic has attracted great interest in virtue of its certain conductivity (3 × 10^7^ S/cm), resistance to corrosion, chemical stability and high permittivity (*ε*). These unprecedented properties make it a promising candidate filler to prepare polymer-based high k nanocomposites [14,15,16]. However, like other ceramic fillers, only when the amount of TiC is high enough can a noticeably high dielectric constant of the composites be obtained, followed by a large dissipation factor and increased conductivity. Furthermore, the high filler loading also brings about other severe problems, such as the phase interfacial defects and poor mechanical and processing properties [17]. Many researchers used hydroxyl groups, coupling agents, surfactants and phosphoric acid to modify the surfaces of nanoparticles to combat this problem [18,19], and these surface modifiers can improve the dispersion of nanoparticles and form a more stable interface layer to suppress dielectric losses of the composites.

Dopamine has recently been appreciated because of its strong adhesion and its easy interaction with various substrates via covalent and noncovalent interactions [2,20]. In addition, aromatic groups and hydrogen bonds are highly polarizable, which helps improve dielectric constants [21,22]. Zhang et al. [23] added Ag nanoparticles coated with polydopamine (PDA) into PVDF, and confirmed the dielectric constant of the obtained composites reached up to a value of 53 at 100 Hz. Thakur et al. [24] presented an environmentally friendly way to form polymethyl methacrylate (PMMA) with dopamine, and the functionalized PMMA films exhibited enhanced dielectric properties compared to pristine PMMA films.

In this study, to reduce the dielectric loss of pristine TiC/TPU, the TiC nanoparticles was surface modified with polydopamine (PDA) to form a core–shell structured nanoparticle PDA@TiC, and the PDA@TiC/TPU composites were prepared by embedding PDA@TiC into a TPU matrix through the solution method. The microstructures of PDA@TiC and dielectric properties of the TiC/TPU and PDA@TiC/TPU composites were investigated in terms of PDA concentration and filler loading. So, the present work aims to provide a deep insight into the effect of PDA and fillers content, and frequency on dielectric and mechanical properties of TiC/TPU composites.

## 2. Materials and Methods

### 2.1. Materials

TPU (1180A, BASF), dopamine hydrochloride (C_8_H_11_NO_2_·HCl) and TiC (40–100 nm) were provided by Zhengfa Plastics Co. Ltd., Alfa, and Guangzhou Hongwu Material Technology Co. Ltd., respectively. *N*,*N*-Dimethylformamide (DMF, AR), Tris (hydroxymethyl)-methyl aminomethane (Tris, AR), HCl (38 wt % aqueous solution), ethanol (AR) and NaOH (AR) were all purchased from the Sinopharm Group. For all experiments, deionized water was used.

### 2.2. Preparation of PDA@TiC and PDA@TiC/TPU Composites

Of the HCl solution 8.5 mL (0.1 mol/L) and 50 mL of the Tris solution (0.1 mol/L) were mixed and diluted with deionized water to obtain 100 mL of a Tris-buffer solution. Dopamine hydrochloride, which is equivalent to 0.1 g dopamine (DA), was added to 50 mL of the Tris-buffer solution, and precisely measured amount of TiC powder (molar ratio of TiC to DA, $n_{\mathrm{TiC}}/n_{\mathrm{DA}}$, were 20:1, 40:1 and 60:1, respectively) was then dispersed into the solution. The mixture was stirred for 48 h at room temperature, and then was vacuum filtered. The filter residue, after being washed with deionized water and ethanol several times, was dried at 40 °C for 12 h, to obtain the modified TiC powder. The prepared PDA@TiC powders were denoted as PDA@TiC−20, PDA@TiC−40 and PDA@TiC−60, respectively.

Of TPU 7 g was added into 10 mL DMF, and the mixture was stirred at 60 °C for 2 h to form a TPU solution. TiC or PDA@TiC−x (where the value of x is determined by the ratio of $n_{\mathrm{TiC}}/n_{\mathrm{DA}}$) powder, which accounts for 1%, 4%, 7%, 10% and 13% of the total weight of the solid materials (TiC and TPU or PDA@TiC−x and TPU), was then added to the TPU solution. The obtained mixture was stirred for 8 h, then heated to 140 °C to evaporate most of the DMF. The resulting composite was further dried at 65 °C in a vacuum oven to remove the residual solvent. The prepared composites with different doping content of TiC or PDA@TiC were denoted as TiC/TPU−1, TiC/TPU−4, TiC/TPU−7, TiC/TPU−10 and TiC/TPU−13 and PDA@TiC−x/TPU−1, PDA@TiC−x/TPU−4, PDA@TiC−x/TPU−7, PDA@TiC−x/TPU−10 and PDA@TiC−x/TPU−13, respectively.

### 2.3. Characterizations

Fourier transform infrared (FT-IR) analyses were performed with a spectrometer (iN10&iZ10, Thermofisher) using KBr pellets in the wave number range of 400–4000 cm^−1^. The Raman spectra were obtained on a Micro Raman Spectrometer (inVia, Renishaw) with an excitation wavelength at 532 nm.

Thermal analysis was performed using a thermogravimetric (TG) analyzer (TGA/DSC1, Mettler Toledo Instruments) to observe the weight changes of the samples as temperature increased.

The morphologies of TiC, PDA@TiC, TiC/TPU and PDA@TiC/TPU were studied via scanning electron microscopy (SEM, S-4800, Hitachi). Before testing, TiC/TPU and PDA@TiC/TPU composites need to be peeled off the copper foils and then brittle broken in liquid nitrogen rapidly. Core/shell-structured PDA@TiC particles were ultrasonic dispersed in ethanol for 10 min, and then dropped on copper mesh for observing their microstructures by using a transmission electron microscope (TEM, H-7650, Hitachi) operating at a voltage of 200 kV.

The dielectric properties were examined in the frequency range of 40–10^7^ Hz at room temperature using an Agilent 4294A impedance instrument (USA). Prior to measurement, the composite was well distributed between two circular copper foils (10 cm in diameter and 10 μm in thickness), and then was loaded into a cylindrical mold (10 cm in diameter) and was hot formed at 200 °C for 15 min with a pressure of approximately 15 MPa. The disk-shaped sample covered with copper foil was then cut into a disk with a diameter of 2 cm for testing. The breakdown voltage of the sample was tested using a HF5013 EHV voltage tester. The breakdown field strength of the sample was calculated using the following formula:

*E* = *U*_b_/*d* (1)

where *E* is the breakdown field strength, kV/mm; *U*_b_ is the breakdown voltage, kV and *d* is sample thickness, mm.

Tensile tests of the samples were conducted with a ZMGI 250 tensile tester (News SANS China), adopting ISO 527-3:1995 as a standard. Dumbbell samples were stretched at a speed of 100 mm/min.

## 3. Results and Discussions

### 3.1. FT-IR Analysis

**Figure 1:**
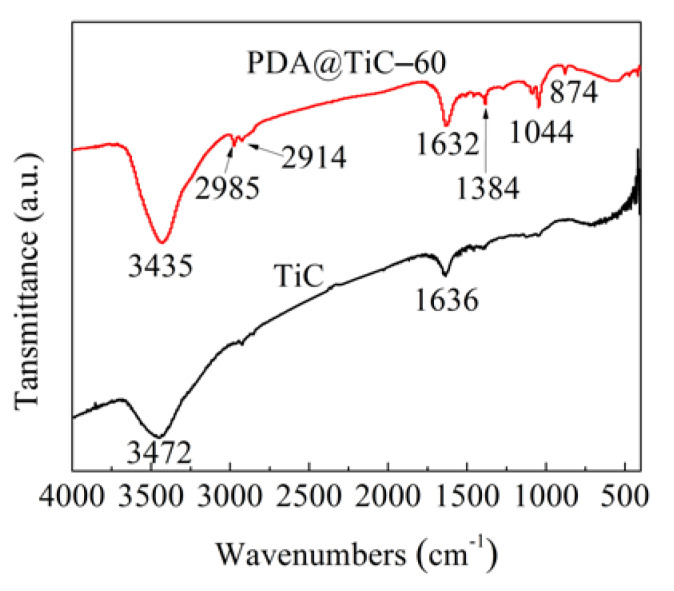
FTIR spectra of TiC and PDA@TiC−60.

FT-IR spectra of TiC and PDA@TiC−60 are shown in Figure 1. The absorption bands at 3472 and 1636 cm^−1^ were caused by residual water absorbed in TiC. Compared with the curve of TiC, the characteristic absorption peaks of PDA appeared inside the curve of PDA@TiC−60. The enhanced peak of 3435 cm^−1^ was caused by stretching vibration of phenolic hydroxyl groups and Ar-NH. The absorption bands at 2985 cm^−1^ and 2914 cm^−1^ were caused by a C-H stretching vibration. The intense absorption features at 1632 cm^−1^ indicate an aromatic ring skeleton (C=C) stretching vibrations and a N-H in-plane deformation vibration. A peak shown at 1384 cm^−1^ was caused by the in-plane vibration absorption of an unsaturated hydrocarbon (=C-H) in the structure of PDA. The new peak at 1044 cm^−1^ was assigned to a C-O stretching vibration, and the last peak at 874 cm^−1^ was attributed to an out-of-plane deformation vibration of N-H. The results demonstrated that a layer of PDA was coated successfully onto surface of TiC.

### 3.2. Raman Analysis

**Figure 2:**
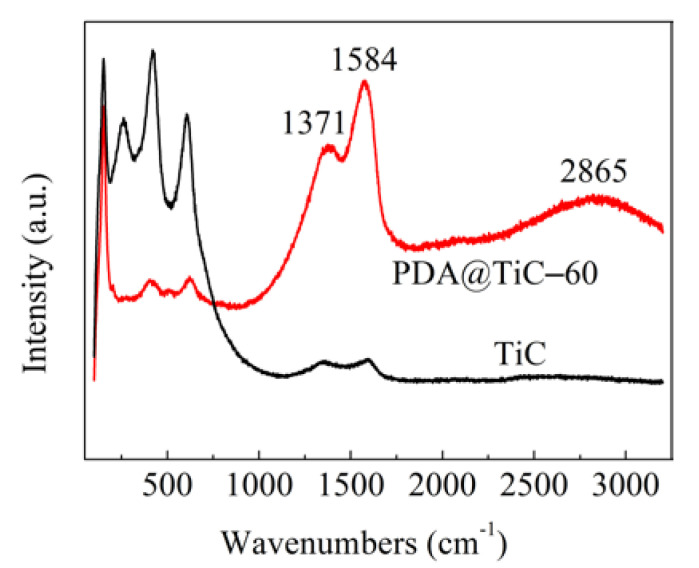
Raman spectra of TiC and PDA@TiC−60.

Raman spectra of TiC and PDA@TiC−60 are displayed in Figure 2. In contrast to pristine TiC the peak intensity was obviously weakened at 200–700 cm^−1^ in the PDA@TiC−60 spectrum. The attenuated signals were caused by PDA coating. The new strong peaks at 1371 and 1584 cm^−1^ appeared after modification, which were ascribed to the deformation of the catechol moiety in PDA. An apparent broad absorption peak was also observed at 2865 cm^−1^, attributable to the unsaturated C-H stretching vibration in the aromatic ring of PDA.

### 3.3. Microstructure Analysis

**Figure 3:**
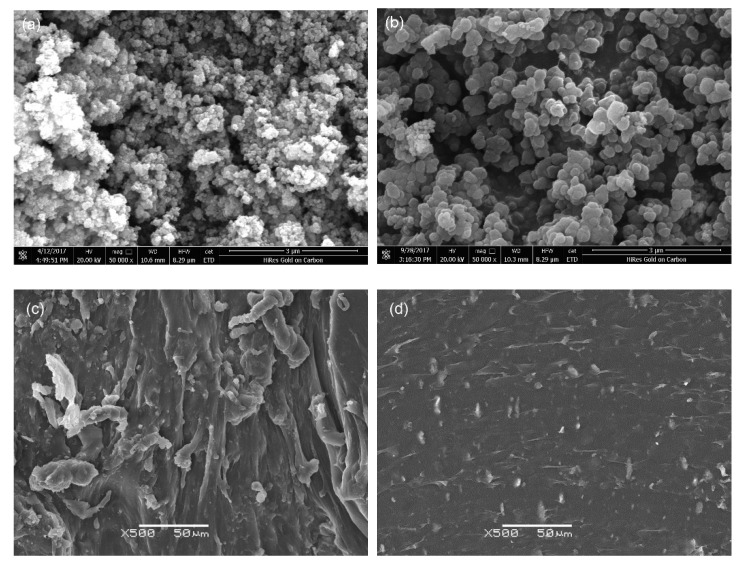
SEM images of TiC (**a**), PDA@TiC−60 (**b**), TiC/TPU−13 (**c**) and PDA@TiC−60/TPU−13 (**d**).

As seen in Figure 3a, the pure TiC particle size was approximately 100 nm, and serious aggregations appeared that result from an electrostatic interaction. Figure 3b shows that the particle size of PDA@TiC−60 was approximately 0.2–1 µm. A smooth surface and relatively regular shape were observed in PDA@TiC−60. The local agglomeration of the nanoparticles can be seen clearly to be effectively blocked by the surface coating. The energy dispersive spectrometry (EDS) analysis results describing TiC before and after treatment are shown in Table 1. There are increasing contents of N and O elements in PDA@TiC−60, indicating PDA has been deposited on TiC particles. Figure 3c,d revealed the dispersion and compatibility between particles and the matrix. It is visible that PDA@TiC−60 particles were more homogeneously dispersed in the composites. No obvious defect was observed at the junction of the two phases that exist in PDA@TiC−60/TPU−13. One reason is ascribed to the strong phase interface adhesion resulting from the PDA coating. The other reason is there are hydroxyl groups in the PDA structure that could form hydrogen-bonding interactions with C-N bonds [25]. Therefore, PDA coating enhances interfacial interactions between fillers and the matrix and improves uniformity of the internal structure of the materials.

**Figure 4:**
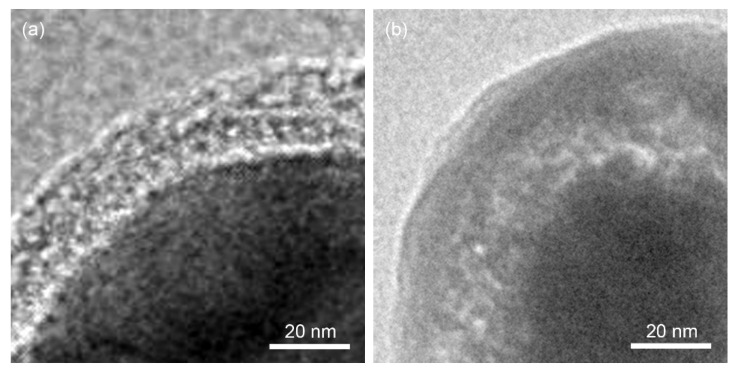
TEM images of PDA@TiC−60 (**a**) and PDA@TiC−20 (**b**).

The TEM images of PDA@TiC particles (Figure 4) revealed that TiC particles were completely encapsulated inside a uniform PDA shell, and the thicknesses of PDA layers were about 20 nm and 40 nm for PDA@TiC−60 and (b) PDA@TiC−20, respectively.

### 3.4. TG Analysis

**Figure 5:**
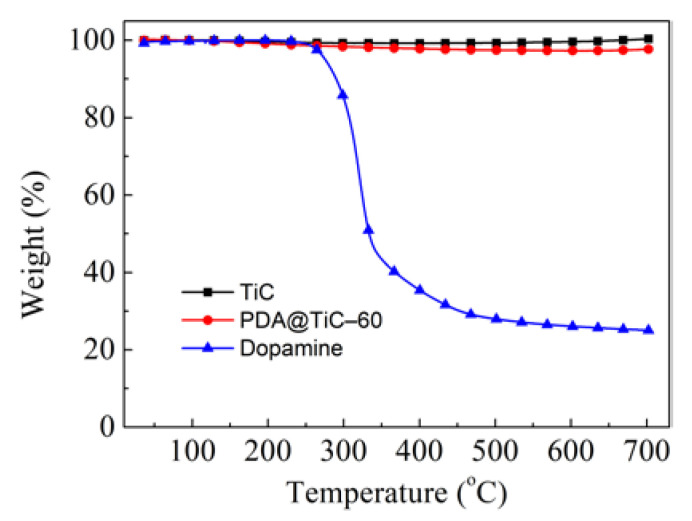
TGA of pristine TiC, PDA@TiC−60 and dopamine.

Figure 5 depicts the results of the TG analysis of TiC particles, PDA@TiC−60 and dopamine, respectively. TiC is a transitional metal carbide with similar properties to metals that have a high melting point and high hardness so its weight was not affected by temperature, while, dopamine is an organic substance that decomposed readily at high temperatures. Dopamine’s weight began to decline dramatically at 250 °C, which is consistent with the loss of features of PDA as reported in [1]. Compared to TiC, PDA@TiC−60 particles also began to lose a small amount of weight at 250 °C. This result further confirms the surface modification TiC with PDA, and corresponded to the results obtained using TEM. The weight loss of PDA@TiC−60 can be measured at approximately 2.8% of its total original weight.

### 3.5. Dielectric Properties

**Figure 6:**
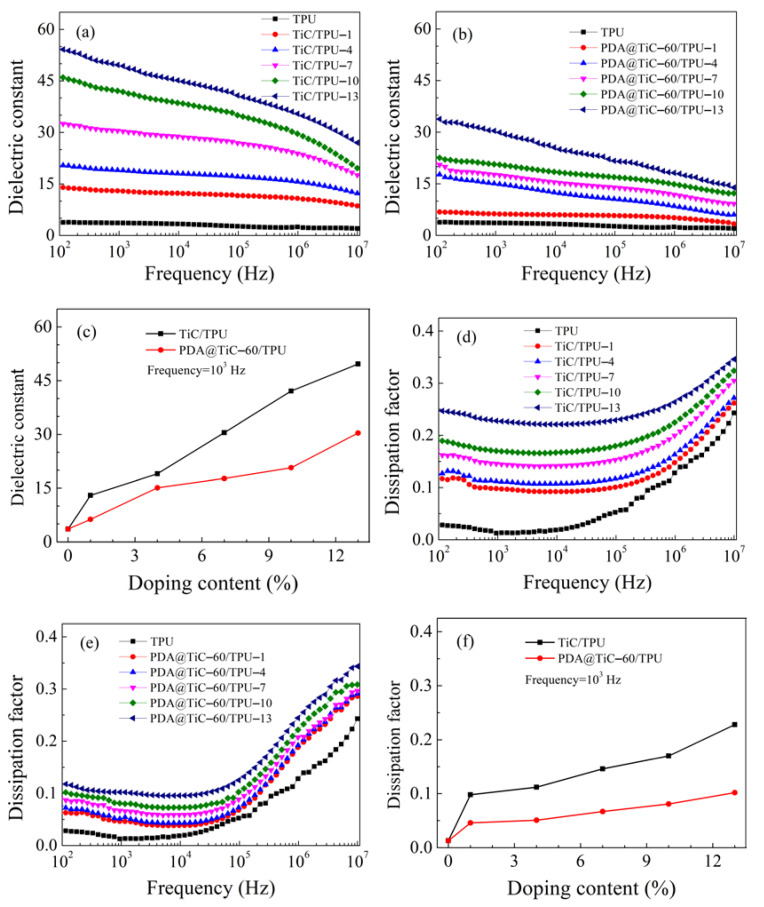
Frequency dependence on the dielectric constant of TiC/TPU (**a**), PDA@TiC−60/TPU (**b**); dielectric constant of TiC/TPU and PDA@TiC−60/TPU with different filler content at 10^3^ Hz (**c**); frequency dependence on dissipation factor of TiC/TPU (**d**), PDA@TiC−60/TPU (**e**) and dissipation factor of TiC/TPU and PDA@TiC−60/TPU with different filler content at 10^3^ Hz (**f**).

The dependence of the dielectric properties of TiC/TPU and PDA@TiC−60/TPU composites are shown in Figure 6 The dielectric constant of pure TPU kept at a low value and shows a weak frequency-dependent behavior. Compared to pure TPU (*ε* = 3.6 at 10^3^ Hz), the dielectric constants of TPU composites with pristine and surface modified TiC were notably increased, and approached to a monotonous increase with the doping content of the fillers in Figure 6a–c. There may be three reasons contributed to this result. Strong interfacial polarization effects would occur at low frequencies according to the Maxwell–Wagner effect; when $\varepsilon_{\mathrm{TPU}}\sigma_{\mathrm{TiC}}\neq \varepsilon_{\mathrm{TiC}}\sigma_{\mathrm{TPU}}$ exist (where $\varepsilon_{\mathrm{TPU}}$ and $\varepsilon_{\mathrm{TiC}}$ are dielectric constants of TPU and TiC, $\sigma_{\mathrm{TPU}}$ and $\sigma_{\mathrm{TiC}}$ are conductivities of TPU and TiC), the effect of interfacial polarization appears in the interface between the PDA shell and the TiC core [26,27,28,29], and the time for the polarization is abundant. The inorganic particles may disrupt the phase separation of TPU, resulting in the disruption of the hydrogen bonding between TPU chains and thus increasing the dipole polarization of the TPU chains under low frequencies [30,31]. Finally, the introduction of TiC provides additional free charges. The dielectric constant gradually decreases with increasing frequencies due to polarization failing to keep pace with the changes of the applied electric field, and a strong dielectric relaxation appears [32], where the composites finally exhibit characteristics related to insulation.

Figure 6e shows that with the increase of PDA@TiC−60 content, the dissipation factors of PDA@TiC−60/TPU exhibited a very slight increase (tan *δ* = 0.05–0.1 at 10^3^ Hz). The change of PDA@TiC−60 doping content had little influence on the dissipation factors. However, the overall value in loss was much smaller than that of the TiC/TPU system (tan *δ* = 0.1–0.23 at 10^3^ Hz), shown in Figure 6d,f.

The dielectric dissipation factor (tan *δ*) measurement formula [33] is:

Tan *δ* = tan *δ*_p_ + tan *δ*_G_ (2)

in which tan *δ*_p_ and tan *δ*_G_ are the relaxation polarization loss and leakage loss, respectively. The tan *δ*_p_ and tan *δ*_G_ can be calculated as:

(3) $$\tan\;\delta_{p}=\frac{\left(\varepsilon_{J}-\varepsilon_{\infty }\right)\omega \tau }{\varepsilon_{J}+\varepsilon_{\infty }\omega^{2}\tau^{2}}$$

(4) $$\tan\;\delta_{G}=\frac{\sigma }{\omega \varepsilon_{0}}\times \frac{1}{\varepsilon_{\infty }+\frac{\varepsilon_{J}-\varepsilon_{\infty }}{1+\omega^{2}\tau^{2}}}$$

where $\varepsilon_{J}$ is static dielectric constant of the medium, $\varepsilon_{\infty }$ is dielectric optical permittivity, *ω* is angular frequency, *τ* is the time constant, *σ* is the conductivity and $\varepsilon_{0}$ is the vacuum dielectric constant.

It can be determined from Equation (4) that when the medium’s conductivity (*σ*) is relatively high a proportionate increase in leakage loss will appear. Loss of polarization is relatively difficult to highlight. The conductivity and agglomeration of TiC make it easy to generate leakage current after energizing, and it results in huge leakage loss. In contrast, the covalent bonds between PDA and TPU tie it down via the -OH, which is free to move. The number of moving charges reduces on the surface of TiC. As a result, the loss caused by leakage current obviously decreases, and the overall dissipation factors value of the composite is effectively cut down. Meanwhile, Equation (4) shows the dielectric loss is proportional to electrical conductivity, which is given expression to the tan *δ* of TPU composites keeping consistent with the content of fillers. In the low-frequency region (10^3^ Hz), tan *δ*_p_ arises from the friction of polarization orientation. In the high-frequency region (10^5^–10^7^ Hz), the tan *δ*_p_ mainly arises from the polymer itself, such as the C-N key polarization. Additionally, the insulating PDA shell on the surface of TiC particles prevents them from direct contact in the matrix, thereby suppressing the loss and decreasing the conductivity to low levels, and the shell thickness of PDA has an effect on the dielectric properties of the composites. So, the dielectric properties of TiC/TPU can be effectively tuned by adjusting the thickness of the PDA interlayer.

**Figure 7:**
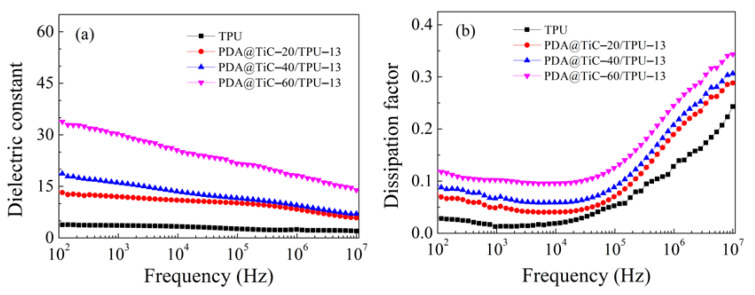

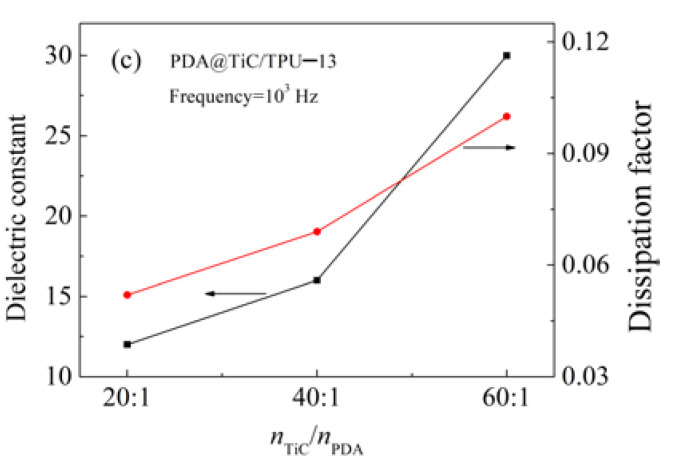
Frequency dependence on the dielectric constant (**a**), dissipation factors (**b**) of TPU and TPU composites with different mole ratio of titanium carbide to dopamine. Dielectric constant and dissipation factors of TPU composites with a different mole ratio of titanium carbide to dopamine at 10^3^ Hz and room temperature (**c**).

Figure 7 presents the effect of the mole ratio of TiC and DA on the dielectric constant and dissipation factor of the TPU composites when the filling quality is set as 13 wt %. As seen from Figure 7a, for the dielectric constant the TPU composites had a similar trend. The dielectric constant of the sample with a higher mole ration of DA (20:1) was 13 at 10^3^ Hz and it remained constant until it was at the frequency of 1 MHz. As shown in Figure 7c with decreasing the mole ratio of the DA mole proportion, the dielectric constant of composites increased from 13 to 31 at 10^3^ Hz. Therefore, it is important to control the shell thickness. According to the Maxwell-Wagner-Sillar (MWS) effect, it can be assumed that the charge carriers were accumulated at the interface of PDA and the TiC near the percolation threshold. The adjacent TiC are considered as parallel electrodes whereas the layer of PDA and PVDF as a dielectric material placed in between electrodes giving rise to several microcapacitors. With an increase in the molar amount of PDA, no changes had taken place in the number of microcapacitors, but the thickness increased (Figure 4) of the dielectric layer, effectively suppressing the electron accumulations at the interface of PDA@TiC and TPU, leading to a low dielectric constant [30].

Figure 7b represents in the low-frequency region (10^2^–10^3^ Hz), with the increase of the PDA coating thickness the loss factor obviously decreased and was kept at a relatively low level (<0.1). There are two possible reasons for this phenomenon. The direct contact and agglomeration between particles among the matrix were effectively prevented by the PDA insulation coating on the surface of TiC, and the leakage current was then suppressed and stopped. The DC (direct current) leakage suppression was more obvious with the increased coating thickness (Figure 7c). Additionally, the interfacial polarization becomes weaker at the same time [34,35,36], which leads to superior performances for the composites.

Dielectric breakdown strength indicates that a material can withstand the highest electric field strength under the action of an electric field to avoid being destroyed. It is calculated by Weibull plots according to the following formula [37]:

*P*(*E*) = 1 − exp(*E*/*E*_0_)^β^ (5)

where *E* is the measured breakdown strength; *P(E)* is the cumulative probability as a function of *E*; *E_0_* is the breakdown strength when the cumulative failure probability is 63.2% and is also used to represent Weibull breakdown strength (Weibull *E_B_*) and *β* is a shape parameter to evaluate the scatter of the measured breakdown strength. According to the IEEE (Institute of Electrical and Electronic Engineers) standard 930–2004, the probability ($p_{i}$) of breakdown is given as:

(6) $$p_{i}=(I-0.44)/(n+0.25)\;\times \;100\%$$

where *i* is the serial number when the breakdown data of *E* are sorted in ascending order and *n* is the number of specimens for each distribution and *n* was 5 in this research.

**Figure 8:**
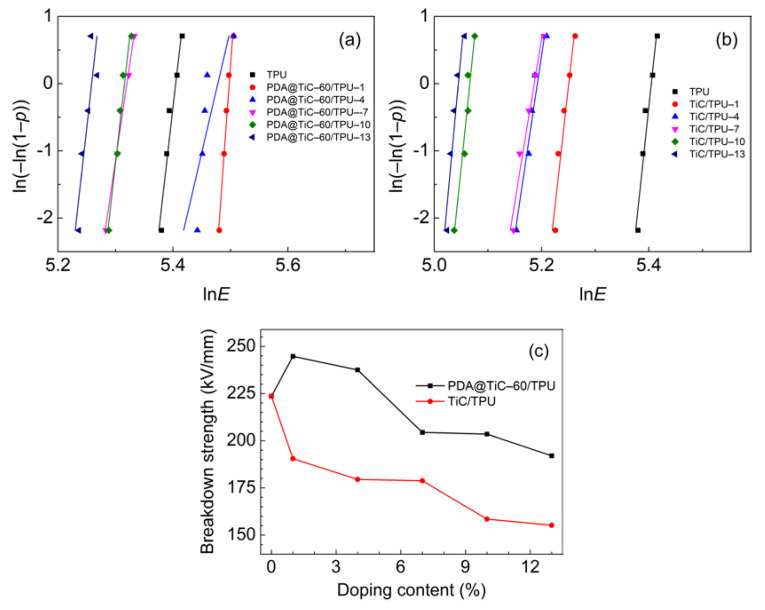
Weibull plots of PDA@TiC−60/TPU (**a**) and TiC/TPU (**b**) and the breakdown strength of PDA@TiC−60/TPU and TiC/TPU with different weight loadings of fillers (**c**).

*E*_B_ of PDA@TiC−60/TPU and TiC/TPU nanocomposites were revealed in Figure 8a,b, respectively. Figure 8c presents the order of *E*_B_ is PDA@TiC−60/TPU−1 > PDA@TiC−60/TPU−4 > TPU > PDA@TiC−60/TPU−7 > PDA@TiC−60/TPU−10 > PDA@TiC−60/TPU−13 > TiC/TPU−x. It indicates that the addition of PDA@TiC−60 particles greatly improved the breakdown field strength of the composites at low filler loading. The particles provided a large number of interfaces in the composite material, and enhanced the interface coupling effect. PDA@TiC−60/TPU was markedly superior to TiC/TPU because of PDA’s weak polar molecular structure and high electrical insulation. The enhanced interfacial interactions between PDA@TiC−60 and TPU imparted the composites with higher dielectric strength because more electrical energy was needed to destroy the samples as compared with the raw TiC/TPU. *E*_B_ decreased with further increasing of two kinds of fillers, which was due to an excess of nanoparticles in the composites producing more empty areas and pores, especially in the interface region of inorganic particles and the polymer matrix [38,39].

**Figure 9:**
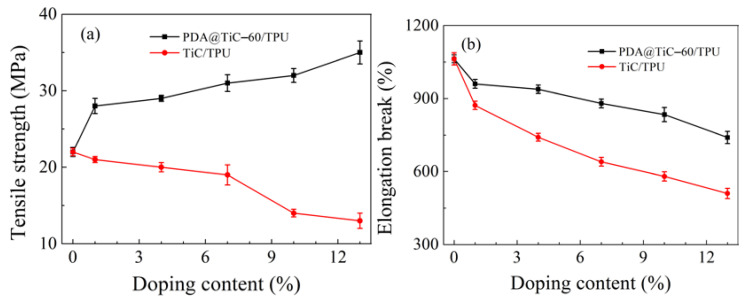
Effect of TiC and PDA@TiC−60 loading amount in TiC/TPU and PDA@TiC−60/TPU on tensile strength (**a**) and strain (**b**), respectively.

Figure 9a shows that the tensile strength of PDA@TiC−60/TPU rose with an increase in the contents of PDA@TiC−60, which was much higher than that observed in pure TPU. This may account for the PDA coating, which provides a strong interface bonding force between TiC particles and the TPU matrix, and improved dispersion of TiC [40,41]. It seems that tensile strength is dependent on interfacial adhesion, where it almost linearly increases with the filler content. The tensile strength of PDA@TiC−60/TPU−13 shows a 169% performance higher than that observed with only TiC. The mechanical properties were unsatisfactory with more added raw TiC, perhaps due to the poor compatibility of the raw TiC with the resin matrix and the agglomeration of TiC particles [42]. Moreover, the phase separation introduces many interfacial defects and plays a negative role in the improvement of the mechanical strength [23,43]. In Figure 9b however, increasing TiC contents would cause an opposite effect in the toughness for two composites. Although PDA@TiC−60/TPU−13 shows the elongation at break (EAB) was lower than that of pure polyurethane it still shows the lowest value of toughness that was up to 740%, which was obviously higher than that observed in TiC/TPU−13 (510%).

## 4. Conclusions

Core–shell structured PDA@TiC composite particles with shell thicknesses about 20–40 nm were successfully synthesized. The dissipation factors of the PDA@TiC−60/TPU remained at a much lower level (tan *δ* = 0.05–0.1 at 10^3^ Hz) when compared with that of pristine TiC (tan *δ* = 0.1–0.23 at 10^3^ Hz) due to the suppressing effect of the PDA shell surrounding TiC. The dielectric constant of the composites with 13 wt % PDA@TiC−60 at 10^3^ Hz could reach up to 31, which was nearly 9 times that of the pure TPU (*ε* = 3.6 at 10^3^ Hz). Increasing the thickness of PDA resulted in better dispersion of PDA@TiC particles in TPU, and slightly decreased both the dielectric constant and the dissipation factor. PDA@TiC−60/TPU composites exhibited enhanced breakdown strength, i.e., 244 kV/mm for TPU with 1 wt % PDA@TiC−60 compared with 190 kV/mm for TPU with 1 wt % TiC, which seemed attributable to the PDA shell. The PDA layer also improved the tensile strength and elongation at break of PDA@TiC/TPU.

## Figures and Tables

**Table 1:** EDS results of TiC and PDA@TiC−60.

Ample	Element Content/wt %				Atomic Ratio/%			
	C	O	N	Ti	C	O	N	Ti
TiC	36.53 ± 3.42	6.93 ± 0.30	0	56.54 ± 3.72	65.22 ± 2.99	9.31 ± 0.05	0	25.47 ± 2.94
PDA@TiC−60	26.37 ± 0.87	8.87 ± 1.02	1.50 ± 0.02	63.26 ± 0.14	50.73 ± 3.37	16.30 ± 4.60	2.47 ± 0.12	30.50 ± 1.10
